# Supplementary material for: Efficient catalyst-free N2 fixation by water radical cations under ambient conditions
Source: Nat Commun. 2024 Feb 20;15:1535. doi: 10.1038/s41467-024-45832-9 (PMC10879522; doi:10.1038/s41467-024-45832-9)
Supplement: Supplementary file 1 — Supplementary Information [file 41467_2024_45832_MOESM1_ESM.pdf]

Supplementary Information

Efficient catalyst-free N_2 fixation by water radical cations under ambient conditions

Xiaoping Zhang[1], Rui Su[2,3], Jingling Li[1], Liping Huang[2], Wenwen Yang[1], Konstantin Chingin[2], Roman Balabin[2], Jingjing Wang[2], Xinglei Zhang[1], Weifeng Zhu[2], Keke Huang[3], Shouhua Feng[3], Huanwen Chen[1,2]*

[1]Jiangxi Key Laboratory for Mass Spectrometry and Instrumentation, East China University of Technology, Nanchang, 330013, P. R. China

[2]School of Pharmacy, Jiangxi University of Chinese Medicine, Nanchang, 330004, P. R. China

[3]State Key Laboratory of Inorganic Synthesis and Preparative Chemistry, College of Chemistry, Jilin University, Changchun 130012, P. R. China

Email: chw8868@gmail.com

Table of Contents

1. Supplementary Note 1: Theoretical calculations……………………………………….3-6
2. Supplementary Note 2: Calculation results in Table 1……………………………………6-7
3. Supplementary Note 3: Calculation of N_2 conversion rate………………………………….7
4. Supplementary Note 4: Calculation results in Table 2……………………………………7-8
Supplementary Figure 1………………………………………………………………….9
Supplementary Figure 2…………………………………………………………………10
Supplementary Figure 3…………………………………………………………………11
Supplementary Figure 4…………………………………………………………………12
Supplementary Figure 5…………………………………………………………………13
Supplementary Figure 6…………………………………………………………………14
Supplementary Figure 7…………………………………………………………………15
Supplementary Figure 8…………………………………………………………………16
Supplementary Figure 9…………………………………………………………………17
Supplementary Figure 10……………………………………………………………..18
Supplementary Figure 11……………………………………………………………..19
Supplementary Figure 12……………………………………………………………..20
Supplementary Figure 13……………………………………………………………..21
Supplementary Figure 14……………………………………………………………..22
Supplementary Figure 15……………………………………………………………..23
Supplementary reference……………………………………………………………..24

1. Supplementary Note 1: Theoretical calculations

Most theoretical calculations were performed using the Gaussian 16 electronic structure programs [1]. ORCA quantum chemistry program package (v5.0.4) was used for fractional occupation number weighted electron density (FOD) analysis [2].

The geometries of reactants, intermediates and products were optimized at dispersion-corrected density functional theory level (DFT-D) with closed- and open-shell virtual orbital-dependent functional as introduced by Grimme (B2PLYP, commonly referred to as B2-PLYP), which uses more than half of exact exchange energy [3,4]. We used the modification of this functional by Martin (B2GP-PLYP) that is expected to be capable of 2 kcal mol^{-1} quality thermochemistry and to be robust toward non-dynamical electron correlation [5]. To estimate the practical uncertainty of our electronic energy differences, we also used the revised version of DSD-PBEP86 functional with Grimme's GD3BJ empirical dispersion correction [6]: relative energy differences ≈ 0.12 eV were observed during this comparison.

Complex Dunning's correlation consistent basis sets of triple and quadruple quality were used to build molecular orbitals: one of Truhlar's triple-zeta "calendar" basis sets (may-cc-pVTZ) was applied for a preliminary optimization [7], while Weigend's modification of Ahlrichs and coworkers quadruple-zeta basis set (def2-QZVPP) was used for the final geometry optimization and vibrational analysis [4]. Fully augmented Dunning's correlation consistent quadruple-zeta basis set (aug-cc-pVQZ) was also used for geometry optimization to test whether basis set limit (BSL) was practically reached [8,9]: negligible differences in B2GP-PLYP and DSD-PBEP86 geometries were observed.

Force constants and the resulting vibrational frequencies were obtained at "very tightly" converged geometries with RMS force threshold of 10^{-6} Hartree $Bohr^{-1}$. Pruned "ultra-fine" grids

with 99 radial shells and 590 angular points per shell were used for all calculations. Vibrational frequencies of all the key species were calculated at the same level of theory to obtain the Gibbs free energies corrections to their electronic energies at 298.15 K in rigid rotor – harmonic oscillator (RRHO) approximation.

Final electronic energies of B2GP-PLYP geometries were calculated at CCSD(T)/CBS level. Valence focal-point analysis of Schaefer [10,11] was applied to reach the complete basis set limit (CBS): Hartree-Fock (HF) energies were extrapolated to BSL by exponential formula, while a two-parameter polynomial formula (X^{-3}) was used for extrapolation of electron correlation corrections (δCCSD(T)). As expected from numerous recent benchmark studies, a difference of ≈ 0.10 eV was observed between CCSD(T)/CBS and B2GP-PLYP/aug-cc-pVQZ data with the exception of $(H_2O)_2^+$ conformations (see below). Because our systems included only period two elements, core correlation shifts and relativistic corrections were estimated to be negligible [12].

The same cannot be said about multi-reference character of our systems with TS being the most problematic case. A set of methods – spin contamination at the UHF level as estimated by the relative deviation from the expected $\langle S^2 \rangle$ value, the low speed of SCF convergence, T_1 diagnostics (>0.035) and FOD analysis (see Supplementary Figure 3) with different density functionals – agrees that our proposed TS for double proton transfer has a large contribution from static (non-dynamical) electron correlation, as regularly happens with biradicals or certain classes of transition-metal complexes [13,14]. So, appearance of many equally important determinants in electronic wave functions is expected. While CCSD(T) with large basis set usually provides reliable reaction energies (accuracy better than 1 kcal mol^{-1}) for single-reference molecules, we cannot reasonably conjecture the same accuracy for this particular molecular system.

This precludes us from drawing a precise conclusion about TS position on the energy diagram.

Highly parameterized Minnesota global hybrid functional from Truhlar with 54% of HF exchange (M06-2X) and τ-dependent meta-gradient-corrected (meta-GGA) functional of Tao, Perdew, Staroverov, and Scuseria (TPSS, commonly referred to as TPSSTPSS) were used to further estimate the uncertainty of quantum chemical calculations [15]. Modification of PBE0/PBE1PBE density functional by setting its exact exchange energy to 7.5% was also tested.

Our choice of virtual orbital-dependent functionals was determined by a comparison with available experimental data in literature [16]. Dinitrogen (N_2) represents an example of complicated electronic system with large static electron correlation (also known as “strongly correlated system” [17]). Therefore, popular DFT functionals (B3LYP, PBE0, TPSS, ωB97X, etc.) are not applicable to N_2, while B2PLYP and its analogues are able to precisely reproduce the experimental data. For example, the bond length and harmonic frequency (ω_e) of the ground-state singlet N_2 (X $^1\Sigma_g^+$) calculated by B2PLYP are 109.78 pm and 2346 cm^{-1}, accordingly. These numbers are highly consistent with the experimental values of 109.77 pm and 2359 cm^{-1}. For comparison, the same results for B3LYP functional are less accurate: 109.01 pm and 2448 cm^{-1}. The dispersion-corrected functional from Head-Gordon (ωB97XD/wB97XD) is even less accurate: 108.72 pm and 2490 cm^{-1}. The same conclusion holds for the triplet N_2 (A $^3\Sigma_u^+$ and B $^3\Pi_g$ states) as well as for the ground doublet state of N_2^+ (X $^2\Sigma_g^+$).

To further test our selection of quantum chemical methodology we have compared our results for HNO ($\tilde{X}$ $^1A'$) with the recent best single-reference WFT estimations available in literature. Due to low stability of HNO molecules, experimental data are not as reliable as needed for a direct comparison [12]. The same trend as with N_2 was confirmed: while B2GP-PLYP/DSD-

PBEP86 geometries were found to be almost indistinguishable (0.1-0.4 pm, 12-15 cm^{-1}) from CCSD(T)/cc-pCVQZ structures of Schaefer, B3LYP/PBE0/M06-2X geometries were not that accurate (1.0-1.3 pm, 56-64 cm^{-1}). The same conclusion holds for a triplet HNO state ($\tilde{a}$ $^3A''$) as well as for a doublet NO (X $^2\Pi$) where comparison with exact experimental data is possible.

Water dimer radical cation was also studied at CCSD(T)/aug-cc-pVQZ level by Schaefer and colleagues [18]. We used these values as a reference point. In case of $(H_2O)_2^+$ hemibonded structure only O..O distance was found to be sensitive to density functional choice: virtual orbital-dependent functionals show 2.6-3.2 pm deviation from the WFT value, while B3LYP/PBE0/wB97XD/PW6B95D3/M06-2X geometries are too loose by 6.0-9.4 pm. Other geometrical parameters are hardly distinguishable for all the methods tested and are close to WFT ones. Unfortunately, inclusion of virtual orbitals into DFT paradigm does not help to reproduce the CCSD(T) relative energies of different $(H_2O)_2^+$ conformations: B2GP-PLYP/def2-QZVPP value of 0.64 eV for energy difference between hemibonded and H-bonded structures is hardly comparable with our value of 0.33 eV at CCSD(T)/CBS level or 0.31 eV from Schaefer.

2. Supplementary Note 3: Calculation results in Table 1

Note that the dimensions of the plate for the discharge needle array were measured as ~ 3.5 cm × 5.5 cm, and thus the given area for the plate was about 19.25 cm^2. However, the effective discharge occurred only at the tip of the needles, which were evenly distributed on the total surface of the plate, *i.e.*, 19.25 cm^2.

Consequently, it could be expected that the water cluster radical cations $(H_2O)_2^{+\bullet}$ would be mainly produced right at the tip of the needle, which was roughly estimated as a small ball [19] with a sharp tip. Using a high-magnification electron microscope, the tip of the tungsten needle observed was spherically shaped, and the diameter of the needle tip was measured to be ≤ 0.06 mm. Take the max value of the diameter (0.06 mm, *i.e.*, (6×10^{-3} cm) for estimation of the surface area, and then according to the ball area (πD^2), the tip surface area was less than 3.14 ×

$6^2 \times 10^{-6} cm^2 = 113.04 \times 10^{-6} cm^2$. Note that only about half area of the tip was exposed to the water vapor for reaction, thus the *S* was estimated as $0.5 \times 113.04 \times 10^{-6}\ cm^2 = 5.65 \times 10^{-5}\ cm^2$. Accordingly, the total effective area surface of the 76 needles in the array was about $4.29 \times 10^{-3}\ cm^2$.

Thus, the actual yield achieved for NH_2OH should be between the $yield_{max} \approx 5.10 \times 10^3$ μg $cm^{-2}\ h^{-1}$ (calculated based on the total effective area of 76 needles) and $yield_{min} \approx 1.14$ μg $cm^{-2}\ h^{-1}$ (calculated based on total plate area). Accordingly, the yield achieved for HNO should be between the $yield_{max} \approx 1.53 \times 10^3$ μg $cm^{-2}\ h^{-1}$ (calculated based on the total effective area of 76 needles) and $yield_{min} \approx 0.37$ μg $cm^{-2}\ h^{-1}$ (calculated based on the total plate area).

The product prices are calculated according to the quotation on Sigma-Aldrich official website. For example, the product price of NH_3 H_2O (~30%) is 30 $ per 500 mL, that is 0.06 $ g^{-1}. The product price of NH_2OH is about 10 $ g^{-1}. The product price of Angeli's (AS) salt (a source to generate HNO) is 30 $ mg^{-1}. As the molar mass ratio of HNO and AS is 1:5 and 1 mol AS salt can release 1 mol HNO in theory, thus, the estimated product price of HNO is about 150 $ mg^{-1}.

3. Supplementary Note 2: Calculation of N_2 conversion rate

Conversion rate = $\frac{\mathrm{C(NH_3+NH_2OH+NO_2^-+NO_3^-+HNO)\ mol/L}}{10\ min} \times \frac{\mathrm{6\ mL}}{\mathrm{1000\ mL/L}} \times 24.5\ \mathrm{L/mol} \times \frac{1}{\mathrm{FR\ L/min}} \times 100\%$, where FR is the flow rate of the gas with 0.1 L min^{-1}. This is based on the assumption that all N_2 fixation products are converted to NH_2OH, HNO, NH_3, NO_2^- and NO_3^-. In detail, C(NH_2OH) = 0.019 mmol L^{-1}, C(HNO) = 0.018 mmol L^{-1}, C(NH_3) = 0.02 mmol L^{-1}, C(NO_2^-) = 0.004 mmol L^{-1}, C(NO_3^-) = 0.007 mmol L^{-1}.

4. Supplementary Note 4: Calculation results in Table 2

The energy cost is calculated according to the equations: the power W = U×I×t (1) and the energy cost = W/n (2), where W is the power, U is the discharge voltage, I is the ion current, t is the reaction time, n is the molar mass of the product. For example, at U = 4.2 kV, I = 0.031 mA, t = 10 min the amount of produced NH_2OH is ca. 3.372 μg (about 0.102 μmol). The power

consumed over 10 min is about 4.2 kV×0.031 mA×1/6 h = 78.12 J. The energy cost is about 78.12 J/0.102 μmol = 770 MJ mol^{-1}.

The product prices are calculated according to the quotation on Sigma-Aldrich official website. For example, the product price of NH_3 H_2O (~30%) is 30 $ per 500 mL, that is 0.06 $ g^{-1}. The product price of NH_2OH is about 10 $ g^{-1}. The product price of Angeli's (AS) salt (a source to generate HNO) is 30 $ mg^{-1}. As the molar mass ratio of HNO and AS is 1:5 and 1 mol AS salt can release 1 mol HNO in theory, thus, the estimated product price of HNO is about 150 $ mg^{-1}. The product price of HNO_3 is about 3 $ per 500g, that is ~0.006 $ g^{-1}. Note that the economy efficiency for the catalytic methods does not account for the price of the catalyst.

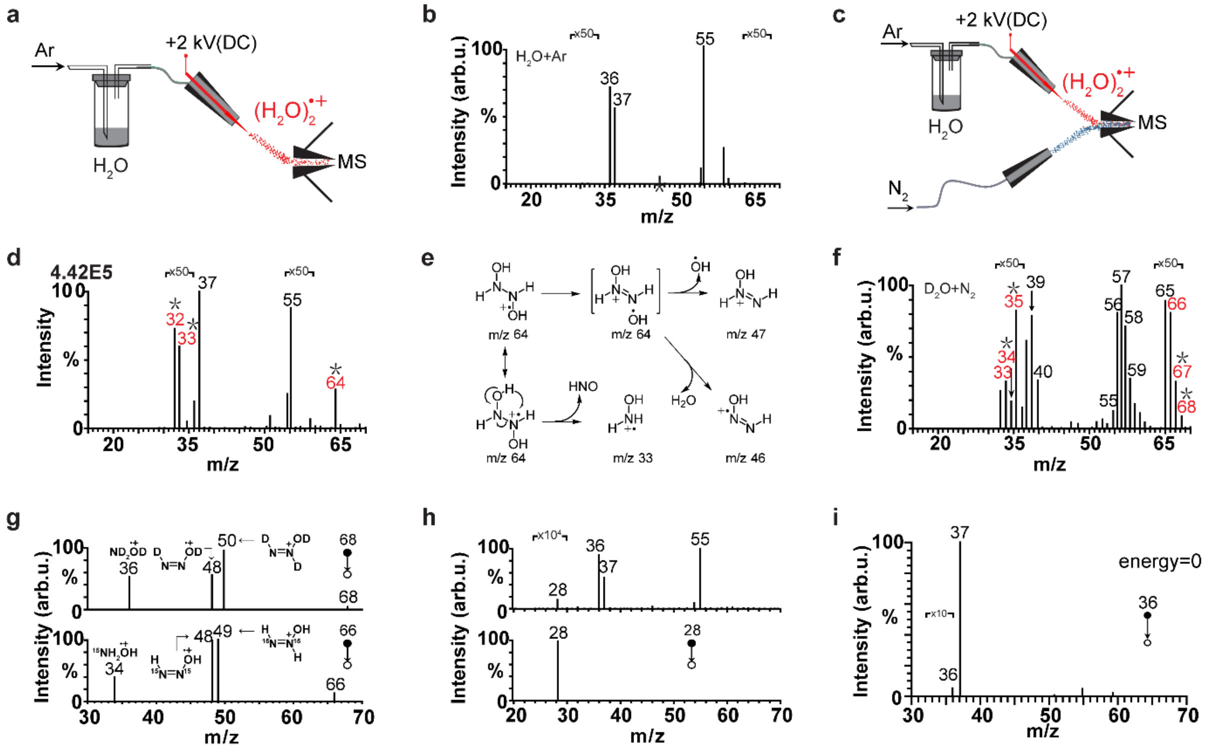

Supplementary Figure 1. Reference experiments with regard to the reaction between N_2 and water radical cations shown in Fig. 1a. a Schematic illustration of the experiment in which N_2 in Fig. 1a was replaced by Ar. The figure is adapted with permission from Supplementary refs. 20-22. **b** Mass spectrum recorded in the experiments shown in Supplementary Fig. 1a was replaced by Ar. **c** Experimental two-channel setup to study the interaction of neutral N_2 with water radical cations by online mass spectrometry. The figure is adapted with permission from Supplementary refs. 20-22. **d** Mass spectrum recorded in Supplementary Fig. 1c. **e** Dissociation pathways of the HONH-HNOH$^{+•}$ intermediate *m/z* 64 formed under the conditions of Fig. 1a. **f** Mass spectrum recorded when H_2O in the experiments shown in Fig. 1a was replaced by D_2O, other experimental conditions being the same. **g** Tandem mass spectra of the HONH-HNOH$^{+•}$-type intermediates revealed in the disproportionation reaction of N_2 with $(H_2O)_2^{+•}$ using different isotopic substitutes: *m/z* 68 (DOND-DNOD$^{+•}$) and *m/z* 66 (HO^{15}NH-H^{15}NOH$^{+•}$). **h** Mass spectrum of $N_2^{+•}$ (corresponding signal at *m/z* 28) in the presence of neutral water vapor in the ion trap. No signals at *m/z* 64 (HONH-HNOH$^{+•}$), *m/z* 32 (HNOH^{+}) or *m/z* 33 ($NH_2OH^{+•}$) could be observed under any experimental conditions tested, even by applying collision energy to the isolated $N_2^{+•}$ ions (isolation width of 5 Da; the activation Q value of 0.4). **i** Isolation of ions at *m/z* 36 inside the ion trap with zero collisional activation energy. Asterisks correspond to the products specific to the reaction between water (D_2O) vapor plasma and N_2.

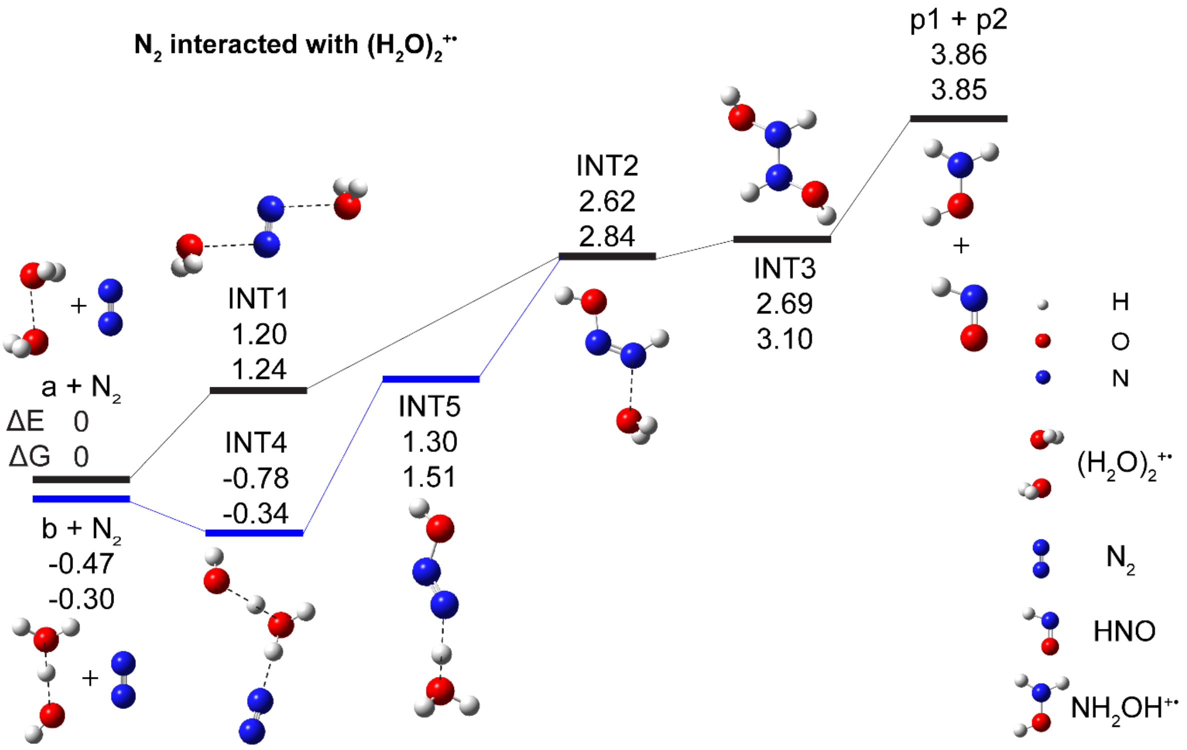

Supplementary Figure 2. The ground-state geometries and energies (in eV at 298 K and 1 atm pressure) of proposed molecular and ionic species involved in the disproportionation reaction N_2 + $(H_2O)_2^{+\bullet}$ → $NH_2OH^{+\bullet}$ + HNO calculated with with CCSD(T) method. a: $[H_2O\bullet\bullet\bullet OH_2]^+$. b: $[H_3O^+\bullet\bullet\bullet OH]$. p1: $NH_2OH^{+\bullet}$. p2: HNO.

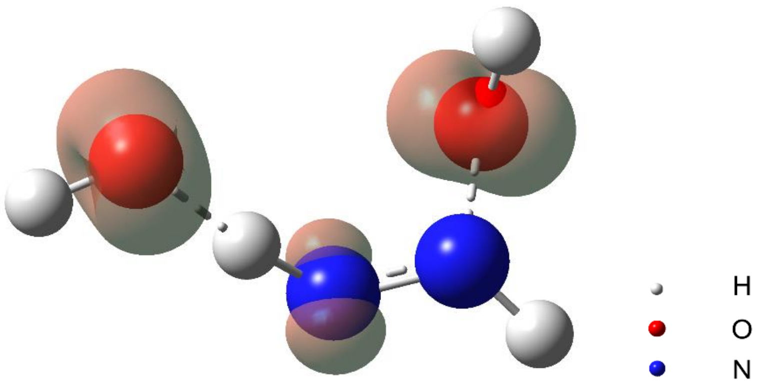

Supplementary Figure 3. Fractional occupation number weighted electron density (FOD) that is plotted as an isosurface at σ=0.005 e Bohr^{-3} level. TPSS/def2-TZVP at 5000 K data shows the "hot" (strongly correlated) electron density regions delocalized on the proposed TS system (N^{FOD} > 0.65).

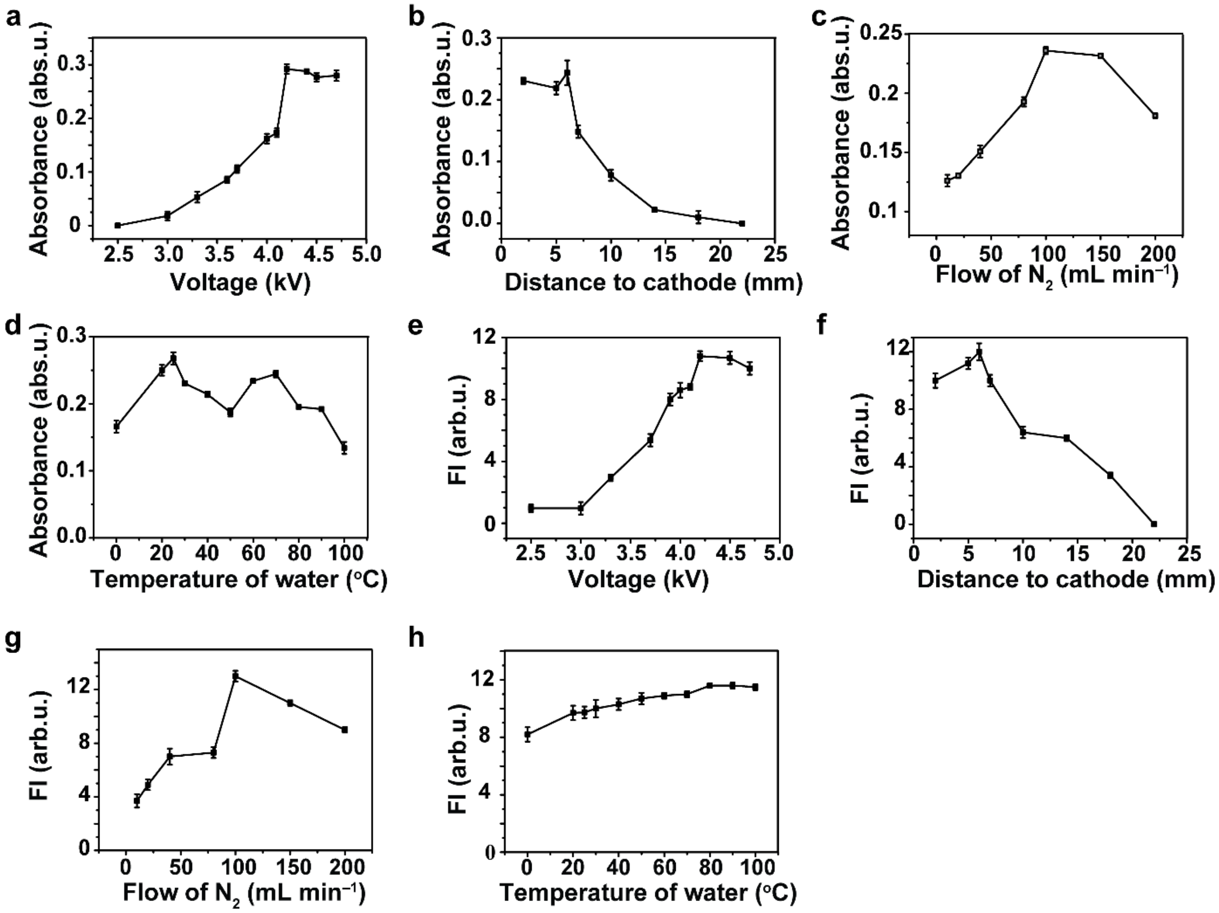

Supplementary Figure 4. The intensity of NH_2OH (a-d) and HNO (e-h) signal depending on experimental parameters: a, e The voltage of needle array. **b, f** Distance from the needle tip to the surface of the solution in cathode. **c, g** Flow rate of N_2. **d, h** Water temperature. The error bars indicate the standard deviation (n=3).

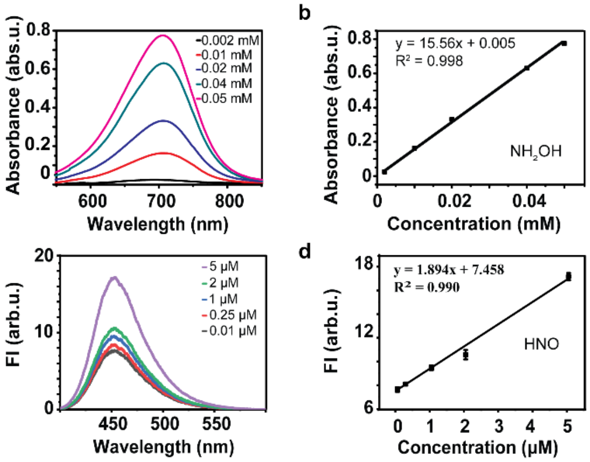

Supplementary Figure 5. Quantification of NH_2OH and HNO by spectrophotometry. a UV-Vis spectra of 8-quinolinol solution reacted with NH_2OH at different concentrations. **b** Calibration curve of NH_2OH. **c** Fluorescence emission spectra of P-CM solution reacted with HNO at different concentrations. **d** Calibration curve of HNO. The error bars indicate the standard deviation (n=3).

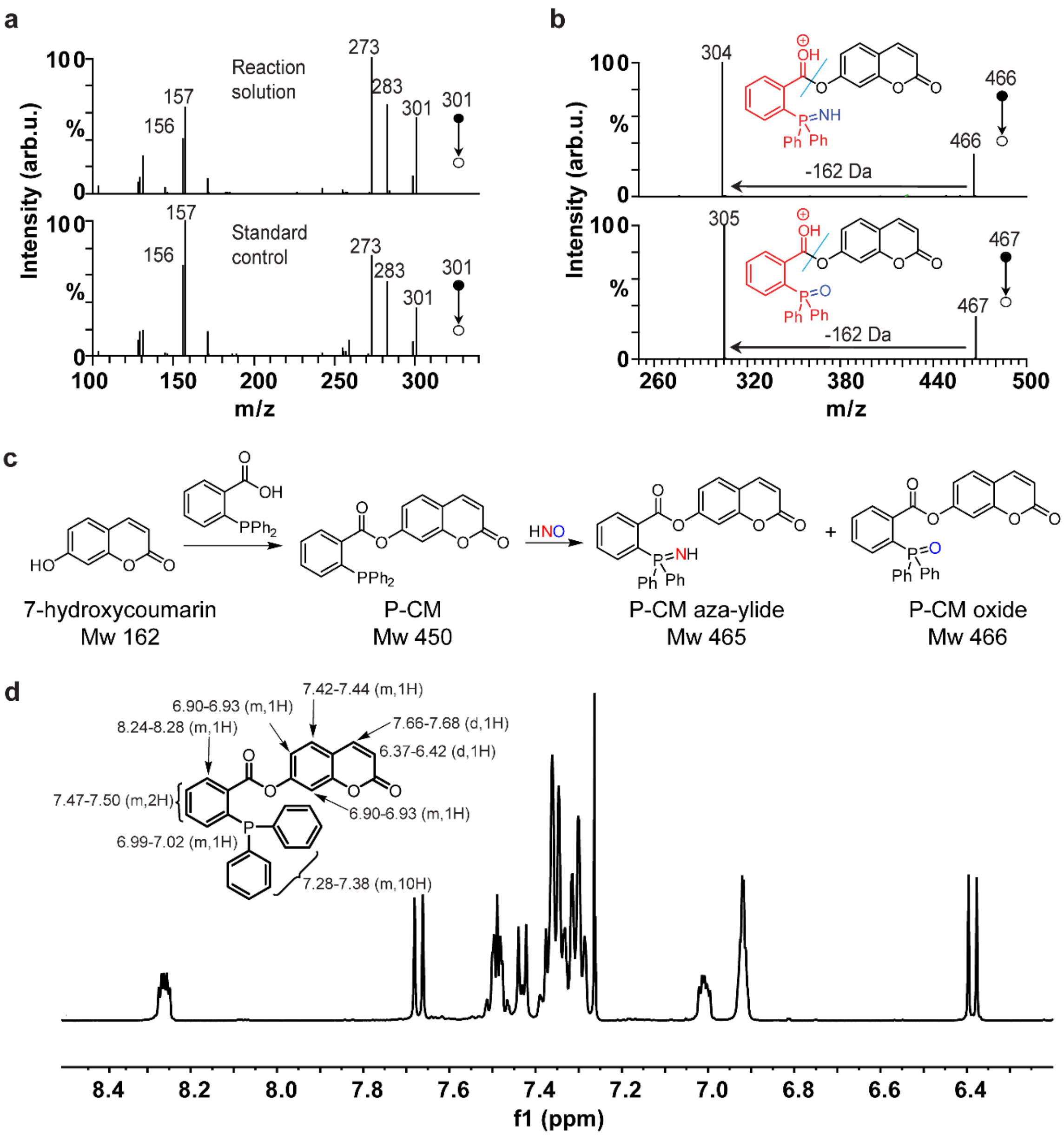

Supplementary Figure 6. Validation of NH_2OH and HNO products by spectral methods. a Confirmation of indooxine formation in the reaction between the collected NH_2OH and 8-quinolinol by tandem MS. **b** Tandem MS characterization of P-CM-NH and P-CM-O products formed by the reaction of P-CM with HNO in the collected reaction mixture. **c** The synthetic route of P-CM probe and reaction process between P-CM probe and HNO [23]. **d** ^1H NMR spectrum of the synthesized P-CM probe, which is consistent with earlier report [23].

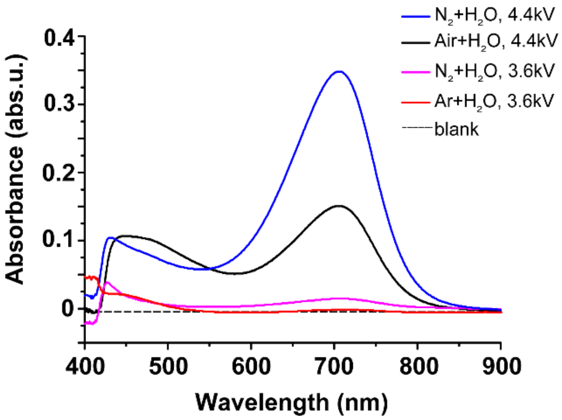

Supplementary Figure 7. Reference UV-Vis spectra recorded for the NH_2OH samples collected using the scaled-up setup shown in Fig. 3a under different conditions. The reference measurements show that the NH_2OH and HNO products were generated specifically due to the reaction between N_2 and $(H_2O)_2^{+\bullet}$. The blank solution was 8-quinolinol solution (6 mL) exposed to humidified Ar under the same experimental conditions for 10 min.

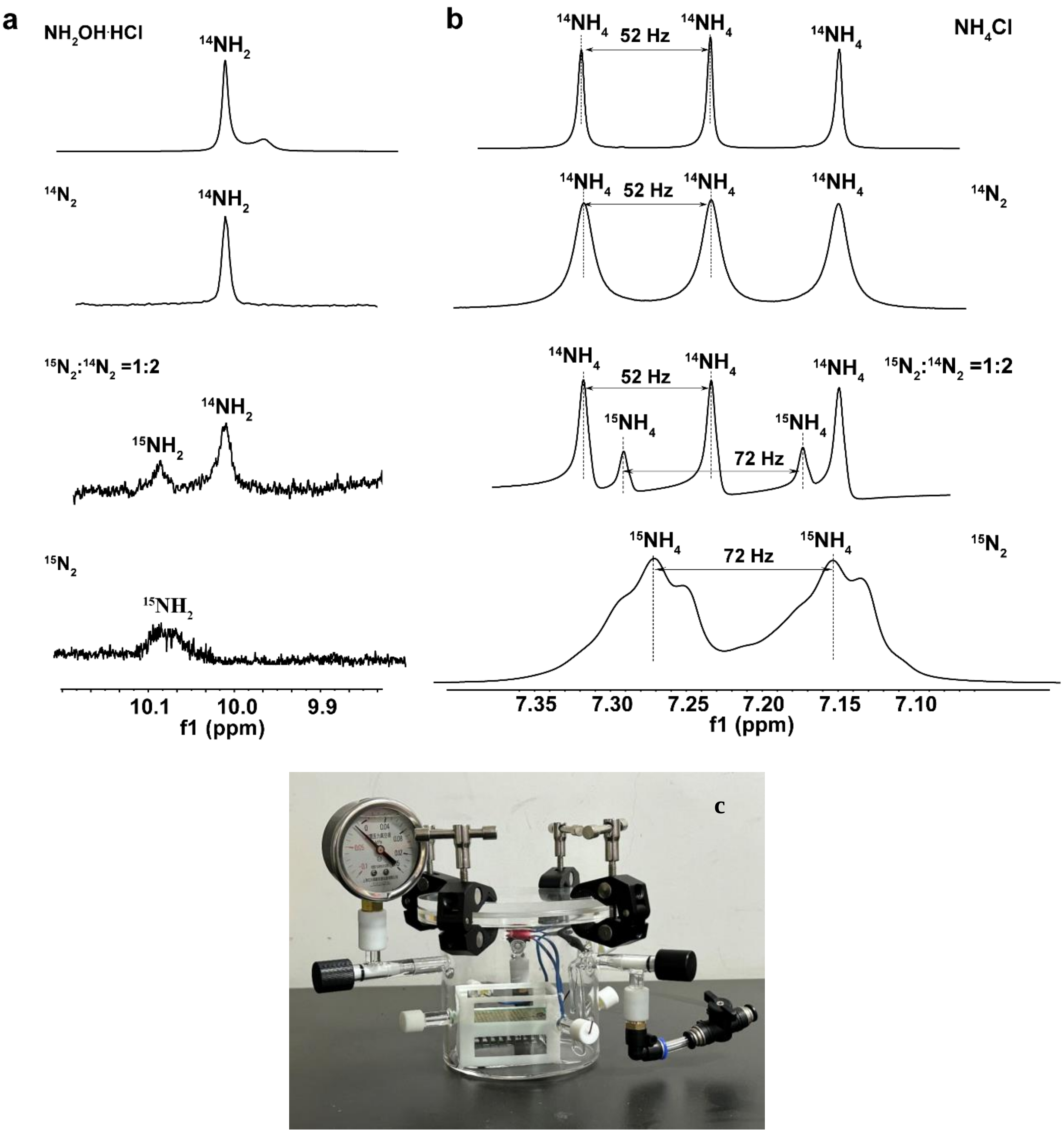

Supplementary Figure 8. NMR spectroscopy of reaction products produced using $^{14}N_2$ and the $^{15}N_2$ gas. a NH_2OH product. **b** NH_4^+ product. **c** Reaction device with enclosure. In order to increase the efficiency of product formation and reduce the cost of isotope $^{15}N_2$ gas, the $^{15}N_2$ gas was introduced into an enclosed container (Supplementary Figure 8c). For comparison, the reference experiment with $^{14}N_2$ gas was done under the same conditions. The ^1H NMR spectrum of $^{14}NH_4^+$ product exhibited a typical triple peak with J = 52 Hz, which matched the peak generated by a standard NH_4Cl. When the mixture of $^{15}N_2/^{14}N_2$ or pure $^{15}N_2$ was used as the feeding gas, a double peak with J = 72 Hz was observed, which was assigned to the $^{15}NH_4^+$ product, similar to that reported by Hu et al. [24].

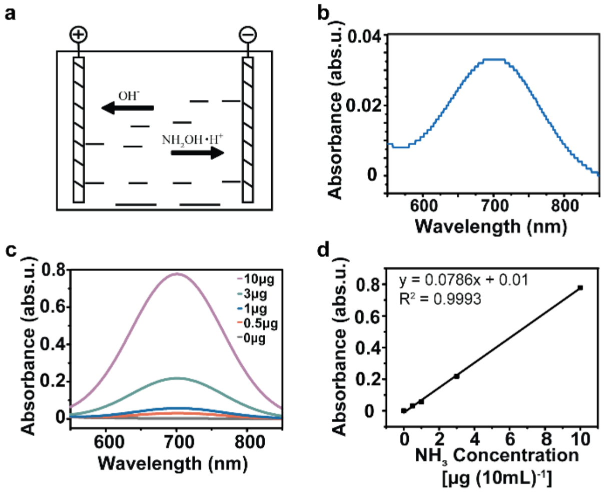

Supplementary Figure 9. Electrochemical reduction of NH_2OH collected during N_2 disproportionation and quantification of the NH_3 product by UV-Vis spectrophotometry. a The device for NH_2OH reduction. **b** Determination of NH_3 produced by the reduction of collected NH_2OH using the indophenol blue method [25]. The concentration of NH_3 product was determined by UV-Vis spectrophotometry of indophenol blue formed upon the reaction of the produced NH_3 with salicylic acid. **c** Reference UV-Vis spectra of indophenol blue formed by the reaction of salicylic acid with aqueous NH_3 at different concentrations (weight per 10 mL of solvent) of NH_4Cl. **d** Calibration curve built based on the data in **c**.

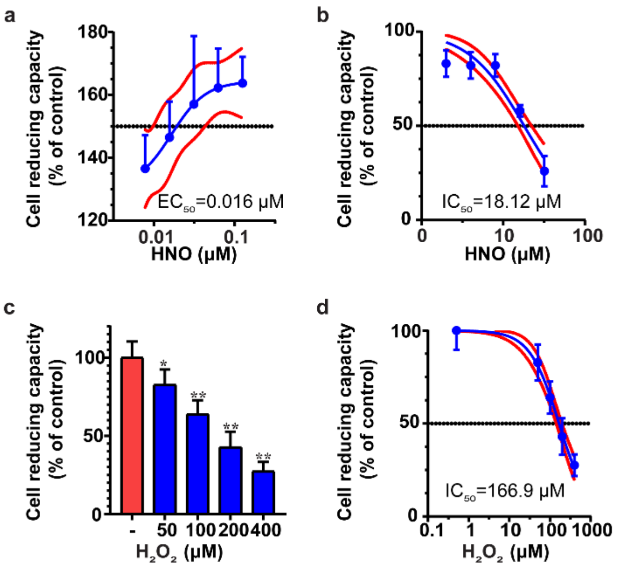

Supplementary Figure 10. The effect of HNO on HT22 cell reducing capacity measured by Cell Counting Kit-8 assay. a HT22 cell reducing capacity as a function of HNO concentration from 0.008 µM to 0.125 µM. The EC_{50} value of 0.016 µM corresponds to HNO concentration at which 50% proliferative effect on HT22 cells is obtained. **b** HT22 cell reducing capacity as a function of HNO concentration from 2 µM to 32 µM. The IC_{50} value of 18.12 µM corresponds to HNO concentration at which 50% damaging effect of HNO on HT22 cells is obtained. **c** Effect of H_2O_2 concentration levels on the reducing capacity of HT22 cells measured by Cell Counting Kit-8 assay. *P<0.05 and **P<0.01 versus control. **d** The reducing capacity of HT22 cells as a function of H_2O_2 concentration levels varied from 50 µM to 400 µM; IC_{50} = 166.9 µM. The error bars indicate the standard deviation (n=3).

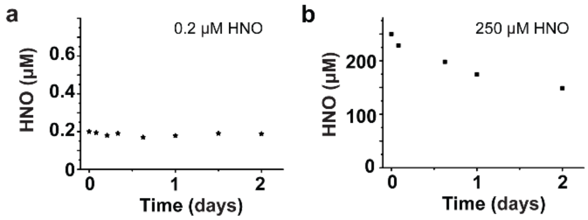

Supplementary Figure 11. The stability of collected HNO aqueous solution: a 0.2 μM HNO; **b** 250 μM HNO.

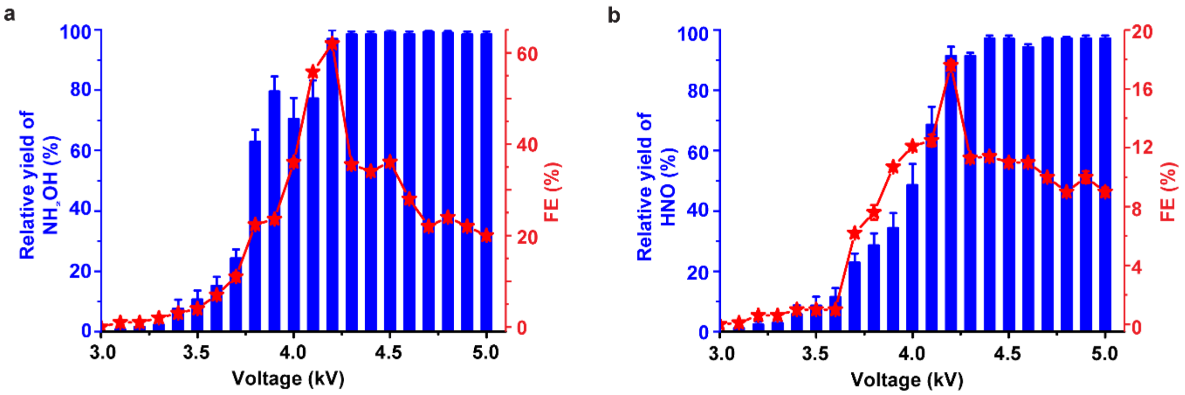

Supplementary Figure 12. Effects of discharge voltage on the relative yields and Faradaic efficiency of ambient disproportionation reaction of N_2 with $(H_2O)_2^{+\bullet}$. a NH_2OH yield and Faradaic efficiency at different discharge voltages. **b** HNO yield and Faradaic efficiency at different discharge voltages. The error bars indicate the standard deviation (n=3).

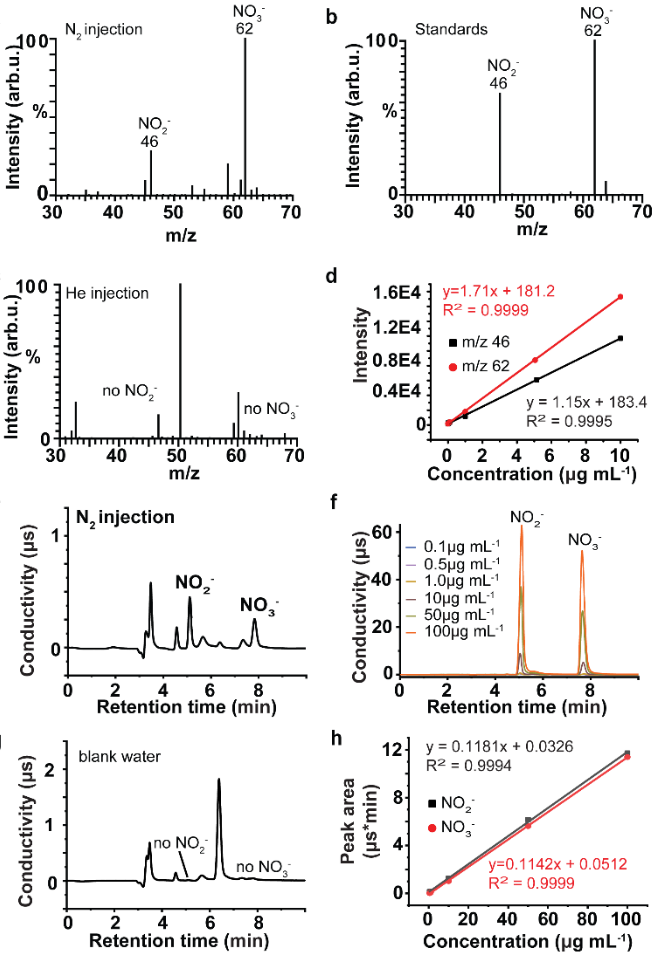

Supplementary Figure 13. Characterization of NO_2^- and NO_3^- products by mass spectrometry (a-d) and ion chromatography (e-h): a online detection of produced species in negative ion detection mode. **b** reference spectrum of standard compounds. **c** blank reference spectrum; **d** calibration curve for the quantitative evaluation of NO_2^- and NO_3^- products. **e** ion chromatogram of the collected reaction gas. **f** ion chromatography of standard compounds. **g** reference blank ion chromatogram. **h** calibration curve**.**

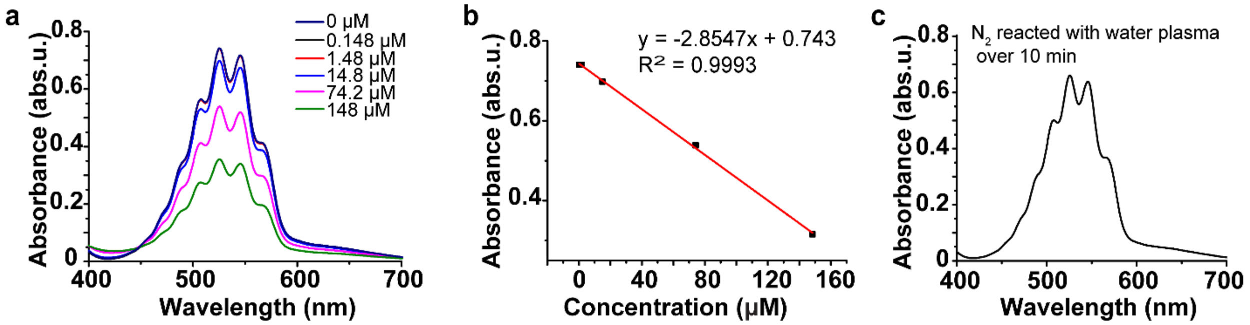

Supplementary Figure 14. Characterization of H_2O_2 product by UV-Vis spectroscopy.

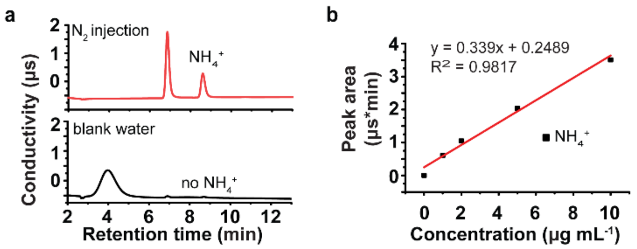

Supplementary Figure 15. Characterization of NH_4^+ by ion chromatography. According to the detection of NH_4^+ by ion chromatography, the concentration of NH_4^+ is ~0.36 μg mL^{-1}. The NH_4^+ production rate was about 0.66 μg cm^{-2} h^{-1}, which was calculated by the formula: Yield = (C×V)/(T×S), where C is the concentration, V is the solution volume, T is the reaction time, S is the area of tip array. Because NH_2OH can convert to NH_3 with nearly 100% efficiency, the ammonia production rate was estimated as ~1.8 μg cm^{-2} h^{-1}.

Supplementary references

1. Frisch, M. J. et al. Gaussian 16. *Revision A.03*, **Gaussian, Inc.**, Wallingford, CT (2016).
2. Neese, F. Software update: The ORCA program system—Version 5.0. *WIREs Computational Molecular Science* **12**, e1606 (2022).
3. Grimme, S. Semiempirical hybrid density functional with perturbative second-order correlation. *J. Chem. Phys.* **124**, 034108 (2006).
4. Becke, A. D., Santra, G. & Martin, J. M. L. A double-hybrid density functional based on good local physics with outstanding performance on the GMTKN55 database. *J. Chem. Phys.* **158**, 151103 (2023).
5. Karton, A., Tarnopolsky, A., Lamère, J.-F., Schatz, G. C. & Martin, J. M. L. Highly accurate first-principles benchmark data sets for the parametrization and validation of density functional and other approximate methods. Derivation of a robust, generally applicable, double-hybrid functional for thermochemistry and thermochemical kinetics. *J. Phys. Chem. A* **112**, 12868-12886 (2008).
6. Kozuch, S. & Martin, J. M. L. DSD-PBEP86: in search of the best double-hybrid DFT with spin-component scaled MP2 and dispersion corrections. *Phys. Chem. Chem. Phys.* **13**, 20104-20107 (2011).
7. Papajak, E., Zheng, J., Xu, X., Leverentz, H. R. & Truhlar, D. G. Perspectives on basis sets beautiful: Seasonal plantings of diffuse basis functions. *J. Chem. Theory Comput.* **7**, 3027-3034 (2011).
8. Dunning, T. H., Jr. Gaussian basis sets for use in correlated molecular calculations. I. The atoms boron through neon and hydrogen. *J. Chem. Phys.* **90**, 1007-1023 (1989).
9. Davidson, E. R. Comment on "Comment on Dunning's correlation-consistent basis sets". *Chem. Phys. Lett.* **260**, 514-518 (1996).
10. Balabin, R. M. Conformational equilibrium in alanine: Focal-point analysis and ab initio limit. *Comput. Theor. Chem.* **965**, 15-21 (2011).
11. Kasalová, V., Allen, W. D., Schaefer Iii, H. F., Czinki, E. & Császár, A. G. Molecular structures of the two most stable conformers of free glycine. *J. Comput. Chem.* **28**, 1373-1383 (2007).
12. Bozkaya, U., Turney, J. M., Yamaguchi, Y. & Schaefer, H. F., III. The lowest-lying electronic singlet and triplet potential energy surfaces for the HNO–NOH system: Energetics, unimolecular rate constants, tunneling and kinetic isotope effects for the isomerization and dissociation reactions. *J. Chem. Phys.* **136**, 164303 (2012).
13. Lee, T. J. & Taylor, P. R. A diagnostic for determining the quality of single-reference electron correlation methods. *Int. J. Quantum Chem* **36**, 199-207 (1989).
14. Grimme, S. & Hansen, A. A practicable real-space measure and visualization of static electron-correlation effects. *Angew. Chem. Int. Ed.* **54**, 12308-12313 (2015).
15. Pan, P.-R., Lin, Y.-S., Tsai, M.-K., Kuo, J.-L. & Chai, J.-D. Assessment of density functional approximations for the hemibonded structure of the water dimer radical cation. *Phys. Chem. Chem. Phys.* **14**, 10705-10712 (2012).
16. Huber, K. P. & Herzberg, G. in *Molecular Spectra and Molecular Structure: IV. Constants of Diatomic Molecules* (eds K. P. Huber & G. Herzberg) 8-689 (Springer US, 1979).
17. Sharpe, S. W. & Johnson, P. M. Triplet Rydberg states in molecular nitrogen. *J. Chem. Phys.* **85**, 4943-4948 (1986).
18. Cheng, Q., Evangelista, F. A., Simmonett, A. C., Yamaguchi, Y. & Schaefer, H. F., III. Water dimer radical cation: Structures, vibrational frequencies, and energetics. *J. Phys. Chem. A* **113**, 13779-13789 (2009).
19. Wang, X.-W., Tao, X.-F., Li, X., He, Z.-Y. & Ren, L.-L. Study on the calibration method of the tip area function of the nanoindenter at super low depth. *Acta Metrol. Sin.* **38**, 593-597 (2017).
20. Zhang, X., Ren, X., Zhong, Y., Chingin, K. & Chen, H. Rapid and sensitive detection of acetone in exhaled breath through the ambient reaction with water radical cations. *Analyst* **146**, 5037-5044 (2021).
21. Zhang, X. et al. Mass spectrometry distinguishing C=C location and cis/trans isomers: a strategy initiated by water radical cations. *Anal. Chim. Acta* **1139**, 146-154 (2020).
22. Yang, W. et al. Determination of C═C Positions of Unsaturated Fatty Acids in Foods via Ambient Reactive Desorption Ionization with Water Dimer Radical Cations. *J. Agric. Food. Chem.* **72**, 845-856 (2024).
23. Mao, G.-J. et al. A highly sensitive and reductant-resistant fluorescent probe for nitroxyl in aqueous solution and serum. *Chem. Commun.* **50**, 5790-5792 (2014).
24. Hu, Y., Chen, G. Z., Zhuang, L., Wang, Z. & Jin, X. Indirect electrosynthesis of ammonia from nitrogen and water by a magnesium chloride cycle at atmospheric pressure. *Cell Rep. Phys. Sci.* **2** (2021).
25. Zhu, D., Zhang, L., Ruther, R. E. & Hamers, R. J. Photo-illuminated diamond as a solid-state source of solvated electrons in water for nitrogen reduction. *Nat. Mater.* **12**, 836-841 (2013).
