# Supplementary material for: Efficient catalyst-free N2 fixation by water radical cations under ambient conditions
Source: Nat Commun. 2024 Feb 20;15:1535. doi: 10.1038/s41467-024-45832-9 (PMC10879522; doi:10.1038/s41467-024-45832-9)
Supplement: Supplementary file 3 — Description of Additional Supplementary Files [file 41467_2024_45832_MOESM3_ESM.pdf]

Description of Additional Supplementary Files

File Name: Supplementary Data 1
Description: The atomic coordinates of the optimized computational models.
